# Prediction of glioma-related epilepsy by Brain Age Index: a multicenter study

**DOI:** 10.3389/fnins.2026.1745461

**Published:** 2026-03-13

**Authors:** Hengyan Liang, Xiaozhou Zuo, Zhang Xiong, Yong Liu, Tao Sun, Xiaochun Jiang, Jiajia Yu, Dongming Liu, Xinru Xu, Jiu Chen, Guangfu Di

**Affiliations:** 1 Department of Neurosurgery, The First Affiliated Hospital of Wannan Medical Collage, Wuhu, Anhui, China; 2 Department of Neurosurgery, The Affiliated Brain Hospital of Nanjing Medical University, Nanjing, Jiangsu, China; 3 Department of Neurosurgery, The First Affiliated Hospital of Bengbu Medical University, Bengbu, Anhui, China; 4 Anhui Digital Brain Engineering Research Center, The First Affiliated Hospital of Wannan Medical Collage, Wuhu, Anhui, China; 5 Department of Radiology, Nanjing Drum Tower Hospital, Nanjing University, Nanjing, China

**Keywords:** brain age, Brain Age Index, glioma, glioma-related epilepsy, MRI

## Abstract

Glioma frequently induces widespread structural and functional alterations extending beyond the tumor site, with epilepsy being one of its most common clinical manifestations. Conventional brain-age models are rarely applied to neurosurgical diseases because focal structural damage violates the assumption of global anatomical integrity. To address this limitation, we propose a novel Brain Age Index (BAI) that integrates bias-corrected brain-age estimations with chronological-age normalization, computed exclusively from non-tumorous brain regions. Using T1-weighted MRI data from 307 glioma patients across three centers and 671 healthy controls, we trained a residual convolutional neural network model for brain-age prediction (mean absolute error, 3.35 ± 4.19 years) and derived the BAI to quantify systemic cerebral alterations. Glioma patients exhibited significantly higher BAI values than healthy controls (*p* < 0.001). Notably, patients with glioma-related epilepsy showed reduced brain-age acceleration compared with non-epileptic patients, suggesting possible adaptive neural reorganization. A combined clinic-radiomic model incorporating BAI achieved an Area Under Curve (AUC) of 0.79 for epilepsy prediction. Collectively, these findings establish the BAI as a promising imaging biomarker for detecting tumor-related cerebral alterations and for enhancing prognostic modeling and functional network assessment in glioma.

## Introduction

Glioma is a common malignant tumor in the central nervous system, comprising 14.2% of central nervous system tumors and 50.1% of malignant cases (Davis, 2018; van den Bent et al., 2023; Ostrom et al., 2022). Gliomas altered both brain structure and function (Yuan et al., 2020; Ainslie et al., 2024; Agarwal et al., 2016; Blumcke et al., 2021). Schiffbauer et al. (2001) findings indicate that, irrespective of grade, intra-axial brain tumors may involve or directly about functional cortex. The extent of involvement of viable functional cortex was greater in low-grade tumors than in high-grade lesions (Schiffbauer et al., 2001). The latest research indicates that the along the perivascular space index of patients with glioma is significantly lower than that of the healthy control group, suggesting impaired lymphatic function (Liang et al., 2025). A study by Zhang et al. (2023) identified that patients with glioma executive dysfunction had increased resting-state activity in the left precuneus and it was negatively correlated with executive function.

Beyond its infiltrative growth and malignant progression, glioma often exerts a profound impact on neuronal excitability and cortical network stability. Consequently, seizures are a common and sometimes early manifestation, collectively referred to as glioma-related epilepsy (GRE). It occurs in approximately 60%–75% of patients with low-grade gliomas and 25%–60% of those with high-grade gliomas (Englot et al., 2016; Erturk Cetin et al., 2017; van Breemen et al., 2007; Yuan et al., 2020) and was thought to be an important clinical indicator of tumor progression (Li et al., 2022a). Fang’s et al. (2022) research indicates that functional connectivity between medial and caudal ventrolateral Brodmann area 6 decreased in the GRE group compared with the non-GRE group. Furthermore, those changes were beyond the local level, which has been demonstrated in the study by He et al. (2024) that the performance of neural networks were decreased in patients with insular gliomas.

To assess structural alterations of the brain in glioma, numerous approaches have been identified. However, conventional imaging methods often missed tumor-induced changes in distant regions and were considered to be insufficient for elucidation of their relationship with functional changes underlying glioma-related epilepsy (He et al., 2024; Almairac et al., 2018). In recent years, brain age models have emerged as a powerful tool for quantifying subtle structural alterations across various neurological disorders, achieving remarkable success in diseases such as Alzheimer’s disease, Parkinson’s disease, and multiple sclerosis (Lee et al., 2022; Cole et al., 2017, 2019; Kaufmann et al., 2019). Previous studies founded that brain aging has been consistently observed to be accelerated in patients with diseases (Lee et al., 2022; Cole et al., 2017, 2019; Kaufmann et al., 2019). Gaser et al. (2013) reported the negative correlation between brain age and cognitive performance, meanwhile higher brain age associated with higher mortality risk. Notably, these disorders typically preserve the overall integrity of brain structure. In contrast, neurosurgical diseases, particularly brain tumors, were characterized by structural destruction. Therefore, directly applying existing brain age models to such cases yielded unreliable results. It highlighted the urgent need to develop novel brain age modeling strategies specifically for neurosurgical patients, enabling a reliable assessment of tumor-related structural and functional remodeling processes. The latest research indicates that a mask-based brain age estimation network can predict acute ischemic cerebrovascular disease, demonstrating the feasibility of mask-based brain age estimation (Zhou et al., 2025).

Beyond structural remodeling, glioma frequently manifests with epilepsy, a major determinant of both clinical management and patient quality of life. Increasing evidence suggests that epilepsy itself induces measurable alterations in brain morphology (He et al., 2024). Patients with newly diagnosed focal epilepsy exhibit atrophy in the anterior thalamus compared with healthy controls, which could reflect early epileptogenic processes (de Bézenac et al., 2024). Brain-age studies further revealed that individuals with epilepsy tended to present older appearing brains than age-matched controls (Hwang et al., 2020). In patients with gliomas, epileptic activities were found to be related to the disrupted topological organization and reduced network efficiency (Fang et al., 2020). However, previous glioma studies not included the assessment of the global cerebral aging and their relations to the pathological processes of gliomas. This gap highlights the need for a unified model capable of analyzing tumor-related structural damages, accelerated brain aging, and the occurrence of glioma-related epilepsy comprehensively.

To address this conceptual and methodological gap, we introduce the Brain Age Index (BAI), a lesion-aware framework designed to extend brain-age modeling to glioma. Rather than relying on the assumption of intact brain anatomy, the BAI focuses on structurally preserved regions to infer systemic and remote alterations induced by tumor growth. This approach provides a new lens for quantifying how glioma reshapes the aging trajectory of the brain and how tumor-induced remodeling contributes to the emergence of glioma-related epilepsy.

## Materials and methods

This study was a retrospective one, and the ethics were conducted through Yijishan Hospital (YH: 2023190), Nanjing Brain Hospital (NH: 2021-KY-124-01) and The First Affiliated Hospital of Bengbu Medical University (BH: 2025KY061X01). Patients gave informed consent upon admission.

### Data preparation

This study was conducted based on data archived from the International Open Access dataset Alzheimer’s Disease Neuroimaging Initiative (ADNI), Yijishan Hospital (YH), Nanjing Brain Hospital (NH) and The First Affiliated Hospital of Bengbu Medical University (BH). The MRI parameters and the advanced filtering terms of the ADNI database are provided in the supplementary document (Supplementary Methods Database, Supplementary Table 1).

**FIGURE 1:**
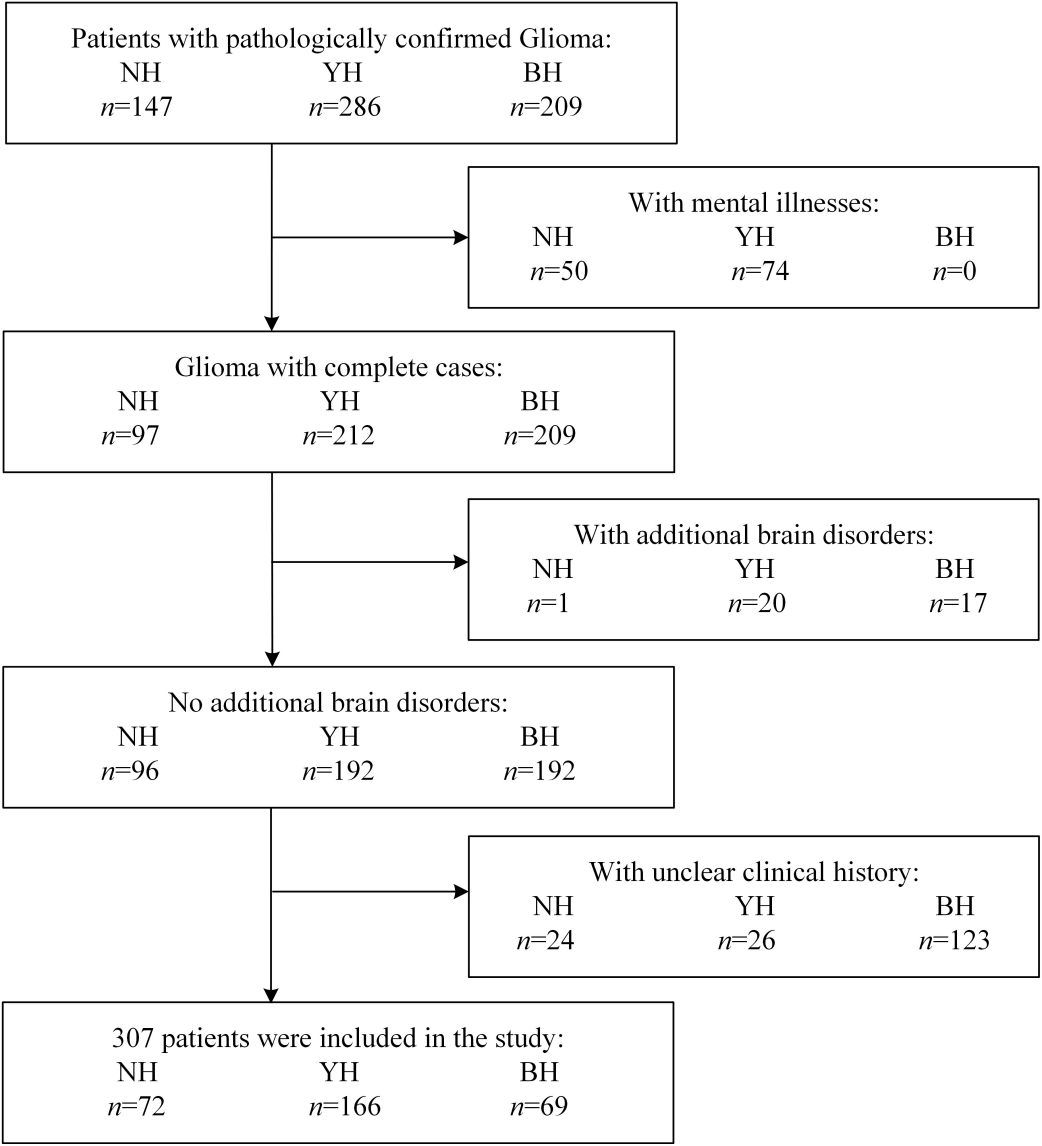
Flow diagram of participant. This figure shows the screening process for glioma patients from four hospitals, BH *n* = 69 (The First Affiliated Hospital of Bengbu Medical University); NH *n* = 72 (Nanjing Brain Hospital of Nanjing Medical University); YH *n* = 166 (Yijishan Hospital). A total of 642 cases were included, and 335 were excluded. Ultimately, 307 cases were included for the prediction of the BAI (Brain Age Index) and GRE (glioma-related epilepsy) models. If a subject had multiple exclusion reasons, it was only counted once.

A total of 307 patients with glioma were enrolled from three centers: BH (*n* = 69), NH (*n* = 72), and YH (*n* = 166). In addition, 671 healthy controls were included from the ADNI database (*n* = 475), NH (*n* = 95), and YH (*n* = 101) (Figure 1). Data were collected from three centers between January 2020 and December 2025, with slight variations in enrollment periods across sites (Center BH: 2023–2025; Center NH: 2024–2025; Center YH: 2020–2025).

To maintain study population homogeneity and analysis reliability, we applied strict exclusion criteria: Co-morbidities: Excluded patients with psychiatric disorders (e.g., depression, anxiety) or other intracranial pathologies (e.g., traumatic brain injury, stroke) to minimize confounding neurobiological effects.

Data Quality: Excluded patients with incomplete medical records, like missing molecular biomarker data (e.g., IDH mutation status) or unclear treatment timelines. This was particularly necessary for the BH cohort, where retrospective data often lacked completeness. This ensured all included subjects had verifiable clinical descriptors for accurate analysis.

In this study, we focus on GRE. GRE refers to a symptomatic, focal epilepsy directly or indirectly caused by brain glioma. According to the coding standards of the International Classification of Diseases, Tenth Revision (ICD-10), glioma typically corresponds to the following primary code: C71.0: Brain tumor (malignant); G40: Epilepsy.

### Data preprocess

All MRI data from NH, YH, and BH were converted to the Neuroimaging Informatics Technology Initiative (NIfTI) format using dcm2niix (v1.0.20241211)^1^ (Li et al., 2016). Intensity inhomogeneities were corrected with N4 bias field correction implemented in SimpleITK (v2.2.1)^2^ (Beare et al., 2018; Yaniv et al., 2018). After grayscale normalization, all images were linearly registered to the MNI152 template and resampled to an isotropic resolution of 2 mmł using the FMRIB Software Library (FSL, v5.0.11)^3^ (Smith et al., 2004). Tumor and peritumoral edema masks were manually delineated by two neurosurgeons (each with 5 years of experience) using ITK-SNAP (v3.8.0)^4^ (Yushkevich et al., 2006) and subsequently reviewed and refined by a senior professor. We calculated the Dice similarity coefficient and intraclass correlation coefficient for the independent segmentation results of two experienced neurosurgeons. The results demonstrate high inter-observer consistency [mean DSC = 0.85 ± 0.04; ICC(2,1) = 0.86, 95% CI: 0.79–0.90] this result was calculated in SimpleITK (v2.2.1)^5^.

### Corrected brain age model

The Brain Age Network (BAN) was developed as a specialized extension of our previously proposed Two-Stage Age Network (TSAN) (Cheng et al., 2021). While the TSAN framework established the baseline for learning high-dimensional correspondence between structural patterns in T1-weighted MRI and chronological age, the BAN was explicitly designed to handle the pathological constraints of glioma patients by focusing on structurally preserved brain regions (Cheng et al., 2021).

**FIGURE 2:**
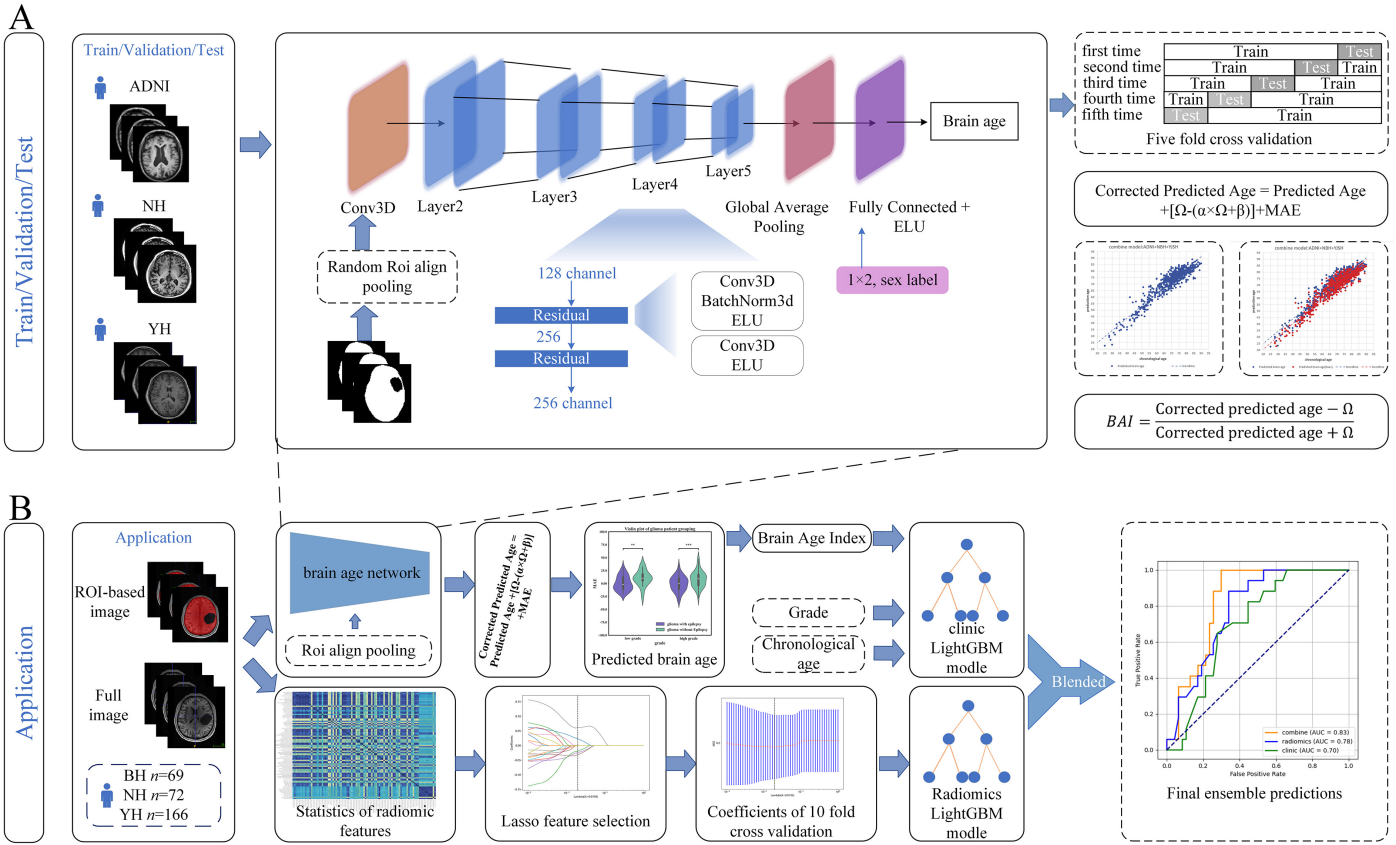
Methodological framework for Brain Age Index computation and its clinical application in predicting glioma-related epilepsy. Panel **(A)** shows that we trained a two-stage residual network brain age prediction model with three databases and performed cross-validation within and between different datasets. Panel **(B)** shows that we used radiomics methods to predict glioma-related epilepsy, used the Brain Age Index model trained in Panel **(A)** to predict brain age, created a clinical model, and fused the clinical and imaging models. BH, The First Affiliated Hospital of Bengbu Medical University; NH, Nanjing Brain Hospital of Nanjing Medical University; YH, Yijishan Hospital. *p < 0.05; **p < 0.01; ***p < 0.001.

Architecturally, the BAN was adapted from the Visual Geometry Group (VGG) network (Simonyan and Zisserman, 2015), a classic deep convolutional neural network architecture known for its use of small (3×3) convolution filters. Specifically, we enhanced the standard VGG backbone by incorporating residual units with short-circuit connections, which allows for more efficient down-sampling through stride-2 convolutions instead of traditional pooling layers (Figure 2). A critical challenge in glioma brain-age estimation is the confounding effect of the lesion itself. To address this, regions corresponding to the tumor and peritumoral edema were excluded using manually delineated masks prior to feature extraction. To process the resulting irregular non-tumor areas, we employed an ROI-Align pooling layer. Unlike standard pooling, ROI-Align accurately extracts feature maps from ROIs of varying sizes and resamples them using PyTorch-based bilinear interpolation, ensuring that the input to the regression head remains consistent despite the heterogeneous lesion volumes.

Given the inherent disparity in age and sex distributions between the glioma and control cohorts, we implemented a re-weighting strategy based on age bins during the training phase. This approach was designed to mitigate the bias introduced by data imbalance on regression learning. Specifically, samples were categorized into age bins, and loss weights were assigned inversely proportional to the bin frequency. It is important to note that these weights were calculated solely within the glioma training dataset, and hyperparameter selection was performed on the glioma validation set to maximize model generalizability.

Model training and inference were implemented in PyTorch (v2.3.0)^6^ (Paszke et al., 2017, 2019) using the Adaptive Moment Estimation optimizer with an initial learning rate of 0.001, weight decay of 1 × 10^6^, β1 = 0.9, and β2 = 0.999. An adaptive learning rate scheduler was employed, reducing the rate by a factor of 0.5 if the training loss plateaued for five consecutive epochs. Training was terminated early if the mean absolute error (MAE) on the validation set showed no improvement for 20 epochs. Further implementation parameters follow the protocols described in our previous work (Cheng et al., 2021).

The dataset was stratified into training (70%), validation (10%), and independent test sets (20%). To ensure robust optimization, five-fold cross-validation was performed on the training subset, while the validation and test sets were held out strictly to ensure unbiased performance evaluation.

To correct systematic bias between predicted and chronological age, a bias-correction function was applied as follows:


Corrected⁢predicted⁢age=Predicted⁢Age+[Ω-(α×Ω+β)]+MAE

where Predicted Age denotes the direct model output, Ω is the chronological age, and α and β are the slope and intercept of the linear bias-correction function, respectively. Incorporation of the MAE term helps maintain adjustment fidelity in the presence of organic lesions.

To quantify the relative deviation of brain aging independent of chronological age scale, a normalized BAI was defined as:


$$B\,A\,I=\frac{\mathrm{Corrected}\,\mathrm{predicted}\,\mathrm{age}-\Omega }{\mathrm{Corrected}\,\mathrm{predicted}\,\mathrm{age}+\Omega }$$

where Ω denotes the chronological age. This formulation normalizes the difference between predicted and actual brain age by their sum, confining the index to the range of −1 to 1. The BAI thus reflects the relative degree of accelerated or decelerated brain aging across individuals, enabling meaningful comparison of brain-age deviations among people of different ages.

### Prediction model of glioma-related epilepsy

To predict glioma-related epilepsy, we constructed an integrated predictive model that combined the BAI, clinical variables (chronological age and tumor grade), and radiomic features extracted from preoperative MRI data. All features were processed under identical training conditions to ensure methodological consistency and guarantee predictive performance.

Feature selection followed a multi-step statistical and correlation-based pipeline. First, the Mann–Whitney U test was applied to identify radiomic features significantly associated with epileptic status (*p* < 0.05; Supplementary Methods, Supplementary Figure 1). To reduce redundancy, pairwise Spearman correlation coefficients were computed, and among features with correlations >0.9, only one representative feature was retained (Supplementary Methods, Supplementary Figure 2). Remaining features were further refined using an iterative redundancy-reduction strategy, preserving maximal descriptive power. Ultimately, 23 features were retained.

Model construction employed the least absolute shrinkage and selection operator (LASSO) regression to identify the most discriminative features (Supplementary Methods, Supplementary Figure 3; Tibshirani, 1996). LASSO regression was implemented using the scikit-learn (v1.5.2) Python package. The optimal regularization parameter (λ) was determined via 10-fold cross-validation using the minimum-criteria rule to minimize validation error (Supplementary Figure 4). Features with nonzero coefficients were used to form the final radiomic signature, with a radiomic score computed as the weighted linear combination of selected features. Multivariate correlation analysis was applied to identify clinical features significantly associated with epileptic status (*p* < 0.05; Supplementary Figure 5).

We constructed a classification model using the LightGBM framework (Ke et al., 2017). Model hyperparameter optimization was performed on the training set through 5-fold cross-validation. Specifically, with a fixed random seed, the training set was further randomly divided into 5 mutually exclusive subsets (folds). In each round of optimization, 4 folds were used as the training subset and the remaining 1 fold as the validation subset to evaluate model performance (Supplementary Table 2). The average performance of this process was used to guide hyperparameter selection. Hyperparameter Tuning Strategy: we adopted a random search strategy to explore the optimal hyperparameter combinations. The defined search space included (but was not limited to): learning rate (0.01, 0.05, 0.1), number of leaves (31, 63, 127), maximum tree depth (5, 7, 10, −1), minimum data in leaf nodes (20, 50), L1 and L2 regularization terms (0, 0.1, 0.01), and feature sampling ratio (0.8, 0.9, 1.0). The optimization objective was to maximize the average AUC of the 5-fold cross-validation. Using the exact same random seed, data splitting strategy, and hyperparameter optimization process (i.e., 5-fold cross-validation combined with random seed), we separately constructed the radiomics model based on radiomics features and the clinical model based on clinical variables. Finally, the out-of-sample predicted probabilities obtained from cross-validation on the training set for these two models were used as intermediate features and input into a logistic regression model to construct the clinical-radiomics combined model, integrating information from different modalities. The final performance of all models was reported based on metrics evaluated on an independent test set.

The operations such as feature selection and model construction are the same in both the radiomics model and the clinical model. In the radiomics model, clinical features were excluded, while in the clinical model, radiomics features were excluded. Since the overall features demonstrated better model performance, subsequent models all used the clinical-radiomics model.

### Statistical analysis

The Kolmogorov–Smirnov test was used to assess data normality. Normally distributed variables are reported as mean ± standard deviation (SD). Group comparisons between patients with glioma-related epilepsy (GRE) and those without epilepsy were performed using the Mann–Whitney U test, and associations among continuous variables were evaluated using Spearman’s rank correlation. Statistical analyses were conducted by scipy^7^ (Virtanen et al., 2020). Verify the robustness of the model through multicenter validation (Supplementary Figure 6).

Model performance was quantitatively assessed using mean absolute error (MAE) and the BAI, with all results reported as mean ± SD. Evaluate the clinical imaging model using receiver operating characteristic curve, sensitivity, specificity, accuracy.

### Data availability

Raw data are available with the consent of the corresponding author.

## Results

### Demographic comparison

A total of 978 participants were enrolled in this study, comprising 307 patients (glioma group) and 671 healthy individuals (control group). The mean age of the overall cohort was 63.3 years (SD = 14.6), ranging from 18 to 90 years. The gender distribution was 565 males (57.8%) and 413 females (42.2%). Within the glioma group, the mean age was 52.3 years (SD = 13.5) with 59.6% males, whereas the control group had a mean age of 68.5 years (SD = 12.1) with 45.2% males. Through the Mann-Whitney U test and the chi-square test, there was a statistically significant difference in age and gender distribution between the two groups of patients (*p* < 0.05). In all models, age and gender were adjusted for, and both age and gender were included as covariates to control for potential confounding effects.

### The analysis of Brain Age Index and mean absolute error

**FIGURE 3:**
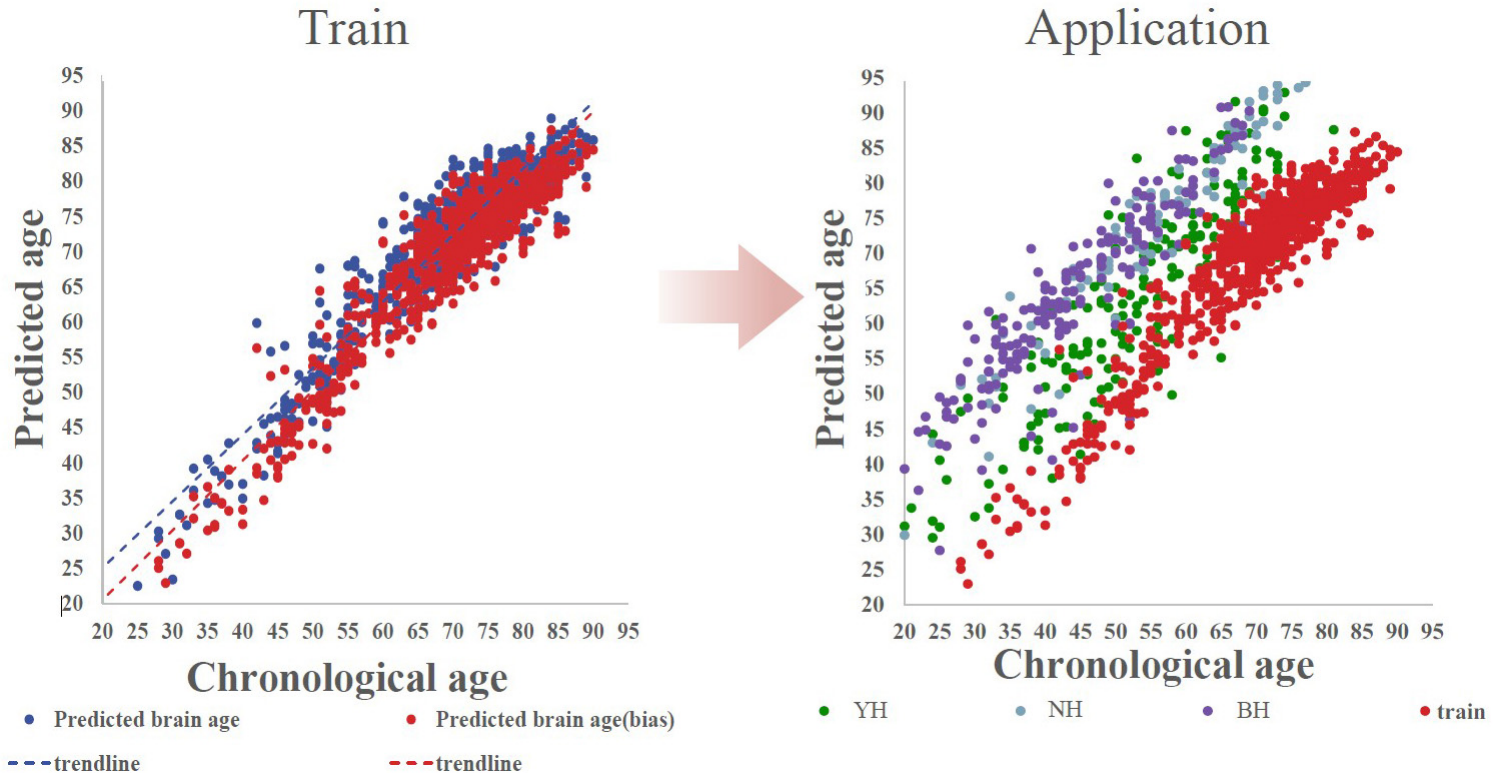
Scatterplots of brain age prediction in normal human data and their application in glioma. Train: the training of bias correction in the normal human model training showed the role of bias correction in the model (*n* = 671). Application: the application of bias correction in the glioma model showed the gap between the brain age of glioma and normal individuals after bias correction. Predicted Age represents the brain age as determined by the model. Predicted Age (Bias) accounts for bias corrections in the brain age prediction. Data is sourced from the Nanjing Brain Hospital of Nanjing Medical University (NH, *n* = 72); The First Affiliated Hospital of Bengbu Medical University (BH, *n* = 69) and Yijishan Hospital (YH, *n* = 166). The “Train” component refers to the bias correction applied by the model (train, *n* = 671).

**FIGURE 4:**
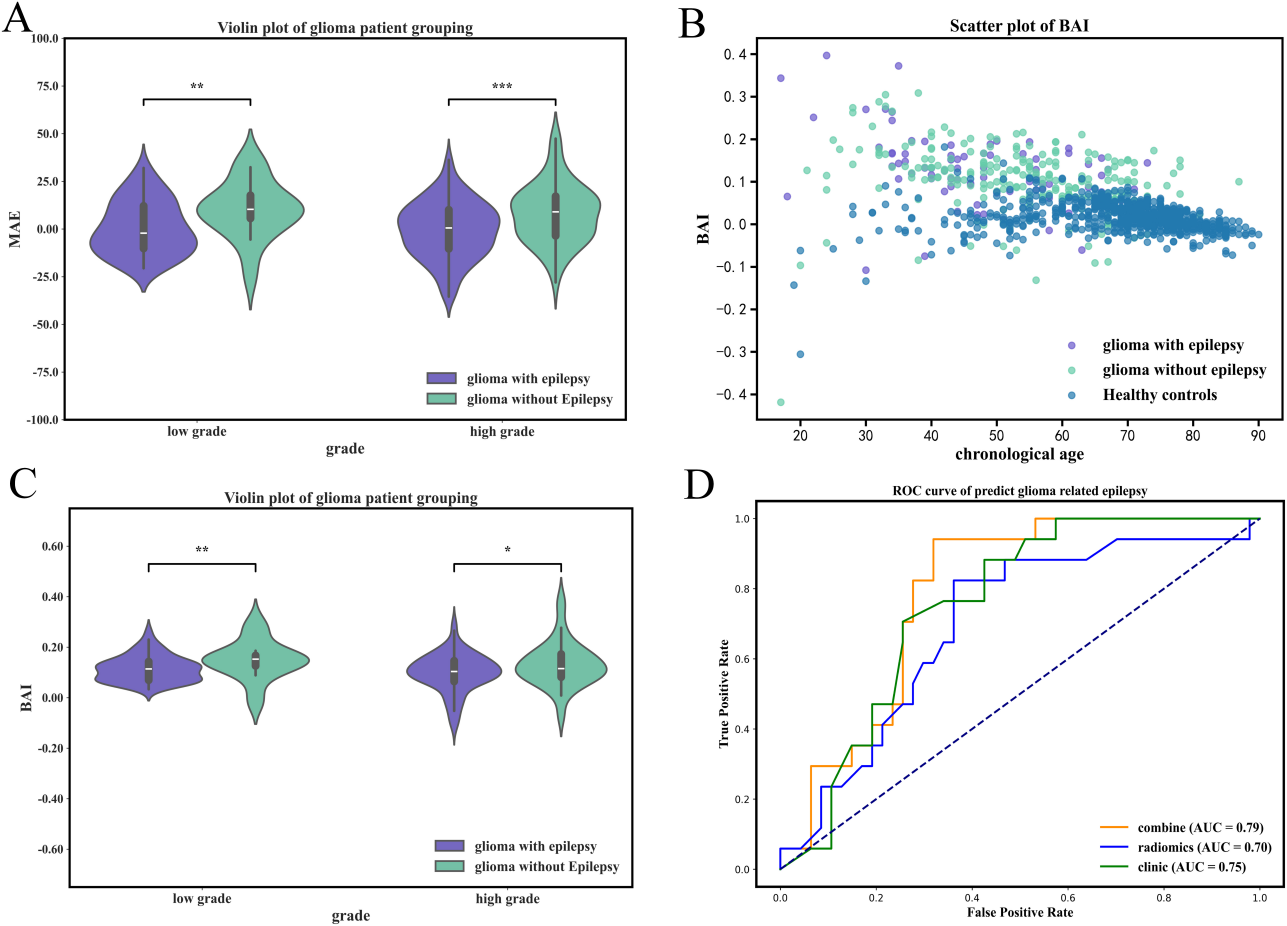
The comparison of healthy controls, glioma with epilepsy and the ROC curve for predicting glioma-related epilepsy. **(A)** Comparison of brain age between patients with glioma-related epilepsy and glioma without epilepsy. **(B)** BAI scatter plot of healthy controls, glioma with epilepsy, and glioma without epilepsy. **(C)** BAI violin plot of gliomas with epilepsy and without epilepsy in different grades. **(D)** Performance of clinic-radiomics models in the prediction of glioma-related epilepsy. *p < 0.05; **p < 0.01; ***p < 0.001.

The model was applied to the clinical cohort comprising glioma patients and healthy controls. MAE was substantially higher in glioma patients (15.84 ± 6.19 years) than in controls (3.35 ± 4.19 years; Mann–Whitney U = 121,269.0, *p* < 0.001) (Figure 3). Patients with GRE (*n* = 92) have significantly lower MAEs (14.98 ± 8.25 years) than those without epilepsy (*n* = 282, 16.63 ± 4.78 years; *U* = 12,854.0, *p* < 0.001), suggesting a relative attenuation of brain-age acceleration in the presence of epilepsy (Figure 4, Supplementary Figures 7, 8).

We further examined the BAI to quantify normalized brain-age deviation across groups. The overall cohort demonstrated a mean BAI of 0.05 ± 0.07. Among glioma patients, those with epilepsy (*n* = 83) showed lower BAI values (0.10 ± 0.08) compared with non-epileptic patients (*n* = 224, 0.13 ± 0.09), whereas healthy controls (*n* = 671) exhibited the lowest BAI (0.02 ± 0.04). Nonparametric tests confirmed significant group differences between epilepsy vs. non-epilepsy (*p* < 0.05), epilepsy vs. controls (*p* < 0.001), and non-epilepsy vs. controls (*p* < 0.001). This difference remains significant after Bonferroni correction (or Benjamini-Hochberg FDR) testing (Figure 4).

### Predicting glioma-related epilepsy

Shapiro–Wilk tests indicated that all BAI values deviated from normal distribution (*p* < 0.05). The BAI values mostly range from approximately −0.3 to 0.4, with the majority clustering between −0.2 and 0.2 and there’s greater variability in BAI values at younger ages compared to older ages.

When classified by tumor grade, low-grade GRE (*n* = 25) displayed significantly lower BAI (0.12 ± 0.05) than non-epileptic patients (*n* = 58, 0.15 ± 0.08; *p* = 0.009), whereas in high-grade GRE (*n* = 58) exhibited reduced BAI (0.10 ± 0.08) compared with non-epileptic cases (*n* = 166, 0.13 ± 0.09; *p* = 0.037).

The imaging model (based on radiomic features) achieved an area under the curve (AUC) of 0.70 (95% CI: 0.67–0.89) on the test set. The clinical model (based on demographic and BAI) yielded an AUC of 0.75 (95% CI: 0.57–0.83). Integration of both features into a clinic-radiomic fusion model (using a Light Gradient Boosting Machine classifier) improved predictive performance. On the training set, this model attained an AUC of 0.85 (95% CI: 0.67–0.89) with an accuracy of 81%, while on the independent test set, it achieved an AUC of 0.79 (95% CI: 0.73–0.92) with an accuracy of 78%. Additionally, on the test set, the fusion model demonstrated a sensitivity of 1.0 and specificity of 0.70. These results demonstrate that incorporating brain-age information enhances the discrimination of glioma-related epilepsy.

## Discussion

In the current study, we developed a novel BAI to assess non-tumorous structural alterations, providing a new framework for quantifying the remote effects of glioma on brain integrity. Our results showed that patients without GRE exhibited higher BAI values than those with GRE, and both groups had higher BAI values than healthy controls. These findings suggest that the BAI may serve as a potential biomarker for predicting glioma-related epilepsy.

### Glioma and epilepsy in the context of brain aging

The concept of brain age provides a quantitative framework to assess deviations from normal aging structures. In the current study, we introduced the BAI, a normalized ratio-based measurement derived from predicted age and chronological age, enabling standardized comparisons across individuals of different ages. By focusing on non-tumor brain regions, our BAI captures global and remote alterations in structural integrity beyond the lesion site.

Our results demonstrated that patients with glioma exhibited significantly higher BAI values and MAE compared with healthy controls, suggesting accelerated structural brain aging associated with glioma-related tissue disruption. Patients with GRE showed relatively lower BAI and MAE values than non-epileptic counterparts. This finding implies that GRE may be linked to a deceleration of apparent brain aging.

There are several mechanisms that can explain this phenomenon. Epileptic activity can induce compensatory or reorganizational changes in cortical and subcortical networks, which may counteract the degenerative effects of the tumor (Blumcke et al., 2021; Li et al., 2022b). Additionally, gliomas associated with epileptic seizures often exhibit distinct molecular or microstructural characteristics, such as lower malignancy grades or slower growth kinetics, which may reflect a less aggressive tumor phenotype (Marcucci et al., 2014; Hathout et al., 2015; Ge et al., 2022).

Furthermore, in patients with diffuse gliomas, increased white matter integrity–specifically, elevated fractional anisotropy–is associated with a higher risk of epileptic seizures (Klein et al., 2025). Patients with temporal lobe gliomas and epilepsy also show a trend of increased mean diffusivity (MD). Seizures in high-grade glioma patients are correlated with improved overall survival, and studies of glioblastoma indicates that patients with a history of epileptic seizures have better long-term survival outcomes (Rilinger et al., 2024; Divé et al., 2025). Studies also suggest that white matter abnormalities detected by diffusion tensor imaging (DTI) in glioma-related epilepsy patients are irreversible, persisting even in patients who remain seizure-free 1 year after surgery, indicating that glioma-related epilepsy may induce underlying structural abnormalities. Brain age estimation was initially calculated based on the volumes of white matter, gray matter, and cerebrospinal fluid. Enhanced white matter connectivity may be a significant factor contributing to the regression of brain age indices in glioma-related epilepsy patients. Moreover, epilepsy patients often seek medical attention early and receive antiepileptic drugs, which may limit secondary structural deterioration and help preserve overall brain integrity. Overall, our findings are consistent with mainstream perspectives, and these factors can explain the observed attenuation of brain age acceleration in glioma-related epilepsy patients. Although epilepsy is associated with brain age and survival outcomes in glioma patients, this does not imply that epilepsy is beneficial. In fact, epilepsy has been shown to promote the proliferation of glioma cells. Epilepsy can also damage or kill neurons, creating space for tumor growth (Ye et al., 1999; Takano et al., 2001).

### Pathology of brain aging in glioma-related epilepsy

Both glioma and epilepsy are clinically refractory conditions. The triggers for glioma-related epilepsy include both excitotoxicity due to “glutamate accumulation” and metabolic reprogramming associated with “IDH mutation,” though their effects on tumor biology are opposite (Ye et al., 1999). Tumor cells overexpress the cystine-glutamate transporter while downregulating EAAT1/2, leading to significantly elevated extracellular glutamate (Ye et al., 1999). While this enhances neuronal excitability and induces epilepsy, it also promotes glutathione synthesis by facilitating cystine uptake, thereby improving antioxidant capacity and favoring tumor growth. In contrast, D-2-hydroxyglutarate, produced by IDH mutation, exerts glutamate-like effects, activating glutamate receptors and triggering epilepsy (Kandil et al., 2010; Andronesi et al., 2012; Elkhaled et al., 2014). However, this mutation itself impairs tumor metabolic adaptability under stress conditions such as hypoxia and nutrient limitation (e.g., restricted reductive carboxylation, continuous NADPH consumption leading to reduced antioxidant capacity, and D-2-hydroxyglutarate inhibiting ATP synthase and affecting energy production), ultimately suppressing tumor progression and resulting in a younger brain age (Marcucci et al., 2014). Therefore, “epilepsy-associated” gliomas often represent molecular subtypes driven by IDH mutation, characterized by slower growth and better prognosis, making epilepsy an indirect clinical phenotype reflecting a milder tumor biology.

### Bias correction for the GRE brain age model

In this study, we observed a significant Brain Age Gap (BAG) between glioma patients and healthy controls (HC). This difference reflects global brain structural damage or accelerated aging caused by the tumor. Since our model was trained on healthy population samples under the assumption that “brain age equals chronological age in healthy subjects,” its application to glioma patients quantifies the degree of deviation of their brain structure from normal physiological states. Notably, to eliminate interference from direct tumor mass effects and edema on feature extraction, we specifically selected “morphologically normal brain tissue” outside the lesion for analysis. This indicates that the observed brain age increase reflects distal damage to whole-brain networks and microenvironments caused by the disease, rather than mere physical destruction by the tumor.

In brain age prediction models, regression toward the mean (where younger subjects are overestimated and older subjects are underestimated) is a ubiquitous physiological system bias. To correct this statistical bias, we improved upon traditional linear correction functions. Conventional correction methods primarily target healthy populations and often mistakenly eliminate structurally significant pathological differences as statistical errors. We introduced the mean absolute error (MAE) as a modulating factor in the correction function, aiming to achieve “statistical bias decentralization” while maximally preserving biologically relevant variations caused by organic lesions. The corrected model not only eliminated systematic skewness associated with chronological age but also maintained stable intergroup differences between patients and controls. This correction strategy ensures that the BAG metric used in subsequent epilepsy prediction analyses excludes systematic errors of the mathematical model while accurately capturing specific brain structural evolution induced by gliomas.

### Predicting GRE: integrating BAI with clinic-radiomics data

We further developed a predictive framework for glioma-related epilepsy that integrates BAI, radiomic signatures, and clinical variables. The imaging-only and clinical-only models achieved moderate performance, whereas their integration into a unified LightGBM-based framework substantially improved predictive accuracy (Ke et al., 2017). This suggests that combining global structural aging markers, BAI, with local radiomic features enhances the identification of epileptogenic potential in glioma.

These results highlight the importance of multidimensional modeling in neuro-oncological prediction tasks. Brain age features reflect widespread, non-local effects of tumors on the brain, whereas radiomic signatures encode spatially localized information on tumor heterogeneity. Their combination thus bridges microstructural and macrostructural representations of tumor–brain interactions, providing a more comprehensive assessment of epileptogenic risk.

As the first study to apply the brain age framework to a glioma cohort, our findings should be viewed as a proof-of-concept. The moderate performance of the predictive model suggests that while a clear biological signal of accelerated brain aging exists in glioma patients, it is not yet sufficient for standalone clinical diagnosis. Rather, brain age gap (BAG) should be considered a novel exploratory biomarker. It may reflect the cumulative neurobiological burden imposed by the tumor, potentially offering insights into a patient’s overall brain resilience and recovery potential–information that is complementary to traditional tumor-centric measurements.

## Conclusion

In this study, we introduced the Brain Age Index (BAI), an innovative imaging biomarker designed to quantify systemic cerebral alterations in glioma patients while circumventing the limitations of conventional brain-age models. By leveraging non-tumorous brain regions for bias-corrected model computation, the BAI effectively captured glioma-induced brain changes and highlighted differences associated with epilepsy-related neural reorganization. Furthermore, the integration of BAI into a clinic-radiomic framework achieved robust prognostic performance for epilepsy prediction, emphasizing its potential to enhance personalized management strategies in glioma. These findings underscore the utility of BAI for advancing our understanding of tumor-related cerebral pathology, facilitating functional network analysis, and improving patient-specific prognostic modeling in neurosurgical diseases.

## Future prospects

Despite the promising results of this study, several avenues for future research remain to be explored to fully translate the Brain Age Index (BAI) into clinical practice for glioma-related epilepsy (GRE): Future studies should focus on longitudinal BAI tracking. Monitoring how a patient’s brain age shifts from the pre-operative phase through post-operative recovery and adjuvant therapy (radiotherapy/chemotherapy) could reveal whether “accelerated aging” is a transient response to seizures or a permanent neuro-structural change. This would be invaluable for predicting long-term seizure freedom. While our current model relies on structural MRI, incorporating functional MRI (fMRI) or Diffusion Tensor Imaging (DTI) could provide a more comprehensive view. Since epilepsy is a network-level disorder, combining structural “brain age” with functional “network age” might significantly enhance the predictive accuracy for seizure recurrence and cognitive outcomes. Glioma management is increasingly driven by molecular signatures (e.g., IDH mutation, 1p/19q codeletion). Future research could investigate the synergistic relationship between molecular subtypes and BAI. Understanding how specific genetic mutations influence the “aging” of the peritumoral and global brain environment could lead to more personalized anti-seizure medication (ASM) strategies.

## Limitations

The current study has several limitations. First, although the multicenter design enhanced data diversity, the overall sample size–particularly within the low-grade glioma and epilepsy subgroups–remains limited, which may restrict the generalizability of the findings and warrants cautious interpretation of subgroup results. Although we addressed the age differences among different groups of glioma patients in the model through weighting, weighting does not equate to complete elimination of confounding, and residual confounding may still exist.

Second, our analyses were confined to T1-weighted structural MRI derived features. Microstructural and functional alterations, such as changes in network connectivities, were not examined but are likely to contribute to the observed aging patterns. Future studies integrating diffusion and functional imaging could provide a more comprehensive understanding of glioma-related brain aging.

Our study did not include a “disease control group” comprising patients with other neurodegenerative conditions (e.g., Alzheimer’s disease or mild cognitive impairment). Consequently, while we demonstrated a significant brain age gap in glioma related epilepsy, we cannot strictly determine the specificity of these structural changes compared to other neurological disorders. Future studies involving diverse clinical cohorts are needed to investigate whether the observed patterns of accelerated aging are unique to epilepsy or share overlapping features with general neurodegeneration. Second, due to the retrospective multicentric design, comprehensive ICD-coded data regarding non-neurological comorbidities (e.g., specific vascular risk factors) were not available for the entire cohort. Although patients with major pathologies were excluded, the potential influence of minor unrecorded comorbidities on brain age cannot be fully ruled out.

Third, the retrospective design limited our ability to incorporate real-time clinical decision-making and longitudinal follow-up. Prospective validation with standardized imaging and clinical protocols will be essential to confirm the clinical applicability and robustness of the proposed brain-age framework in real-world neurosurgical settings.

## Data Availability

The raw data supporting the conclusions of this article will be made available by the authors, without undue reservation.

## Footnotes

1
https://github.com/rordenlab/dcm2niix

2
https://simpleitk.readthedocs.io/en/master/

3
https://fsl.fmrib.ox.ac.uk/fsl/fslwiki

4
https://www.itksnap.org

5
https://simpleitk.readthedocs.io/en/master/

6
https://pytorch.org

7
https://scipy.org/
