# Supplementary material for: Prediction of glioma-related epilepsy by Brain Age Index: a multicenter study
Source: Front Neurosci. 2026 Mar 13;20:1745461. doi: 10.3389/fnins.2026.1745461 (PMC13021608; doi:10.3389/fnins.2026.1745461)
Supplement: Supplementary file 1 [file Data_Sheet_1.docx]

# Supplementary information

## Methods

### Dataset

### Image preprocessing

### Statistical analysis

## Results

### Brain age training model five-fold cross-validation

### Whole brain brain age prediction

### Whole brain glioma with epilepsy brain age

## **Supplementary table legends**

### Table S1: Five-fold cross-validation

## **Supplementary figure legends**

### Figure S1. Statistics of radiomic features

### Figure S2. Correlation coefficient visualization

### Figure S3. Path plot of lasso coefficients

### Figure S4. Lasso regression analysis was used for cross-validation

### Figure S5. Clinic multivariate analysis

### Figure S6. Multicenter cross-validation of brain age

### Figure S7. Whole brain glioma brain age prediction (After correction of bias)

### Figure S8. Comparison of brain age between patients with glioma related epilepsy and glioma without epilepsy

## **Methods**

### **database**

**Imaging parameters**

| **Parameter** | **Nanjing Brain Hospital** | **Yijishan Hospital** | **First Affiliated Hospital of Bengbu Medical University** |
| --- | --- | --- | --- |
| **MRI Scanner** | Siemens Verio 3.0 Tesla | GE Signa HDXT 1.5 Tesla | GE Discovery MR750 3.0 Tesla |
| **Repetition Time (TR)** | 1900 ms | 2000 ms | 1900 ms |
| **Echo Time (TE)** | 2.48 ms | 8.30 ms | 2.93 ms |
| **Flip Angle** | 9 degrees | 150 degrees | 9 degrees |
| **Echo Train Length** | 1 | 8 | 1 |
| **Imaging Frequency** | 123.155528 MHz | 63.663378 MHz | 123.246053 MHz |
| **Slice Number** | 176 slices | 17 slices | 160 slices |
| **Slice Thickness** | 1 mm | 6.5 mm | 1 mm |
| **Number of Excitations (NEX)** | 1 | 1 | 1 |
| **Rows** | 256 pixels | 512 pixels | 512 pixels |
| **Columns** | 256 pixels | 408 pixels | 448 pixels |
| **Pixel Spacing** | 1 x 1 mm | 0.5 x 0.5 mm | 0.48828125 x 0.48828125 mm |
| **Field of View (FOV)** | 256.00 x 256.00 mm | 230.00 x 183.28 mm | 250.00 x 218.75 mm |
| **Voxel Size** | 1 x 1 x 1 mm | 0.5 x 0.5 x 6.5 mm | 0.5 x 0.5 x 1 mm |

Table 1. Imaging parameters in three hospitals

The Alzheimer's Disease Neuroimaging Initiative (ADNI) database is a large, multicenter, long-term publicly available database. The ADNI database contains a large number of clinical data, imaging data and biomarker data from patients participating in the ADNI study. We used advanced filtering in the adni database to select 475 data for inclusion in this study. Advanced screening criteria: "ADNI1", "ADNI2", "ADNI3", "ADNI4", "ADNI GO", "T1-MPRAGE", "CN".

Detailed inclusion criteria and exclusion criteria is in main text. (Figure 1.)

### **image preprocessing**

(1) We converted MRI data from DICOM to NIfTI format with dcm2niix (<https://github.com/rordenlab/dcm2niix>)^[1]^. MRI data in the public dataset are already in NIfTI format; no further format conversion is required. (2) Using a python SimpleITK (https://simpleitk.readthedocs.io/en/master/) software package for N4 interchange bias field correction^[2]^. (3) Based on clinical data acquisition parameters, we use the FMRIB software library (FSL) (https://fsl.fmrib.ox.ac.uk/fsl/fslwiki), after all the image grayscale normalization with linear registration aligned all data to MNI152 1-mm standard T1 structure, And resampled to 91×109×91 with an isotropic spatial resolution of 2mm3.^[3]^ (4) The tumor was delineated layer by layer using ITK-SNAP by a physician with 5 years of neuroimaging experience^[4]^. This was then confirmed and modified by a neurologist with a senior professional title using the same software.

### **Statistical analysis**

Kolmogorov-Smirnov test was used to determine whether the data were normally distributed. Generally, distribution data are described as mean± standard deviation (mean±SD). The independent sample t-test was used to compare the brain age differences between glioma related epilepsy and those without epilepsy. Spearman correlation analysis was used to measure the correlation between continuous variables. SPSS22.0 was used for statistical analysis. Mann-Whitney U test was used for statistical test and feature selection of radiomics features. All features are extracted with an in-house feature analysis program implemented in Pyradiomics（http://pyradiomics.readthedocs.io）^[5]^.


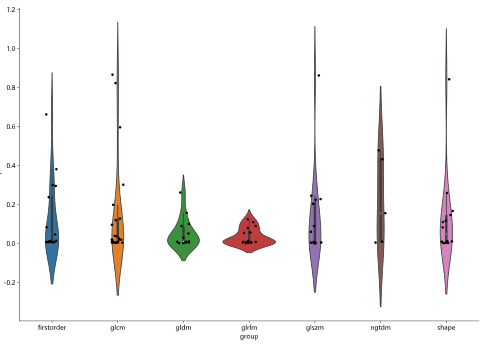


Figure S1. Statistics of radiomic features


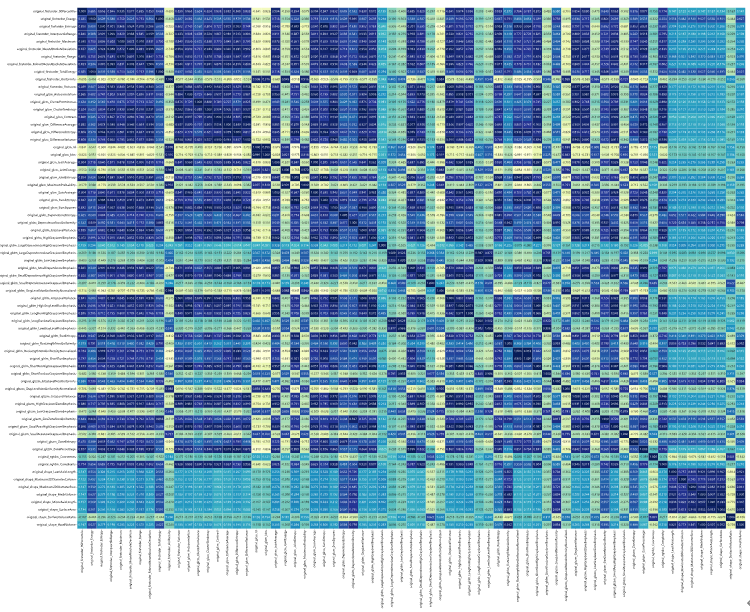


Figure S2. Correlation coefficient visualization


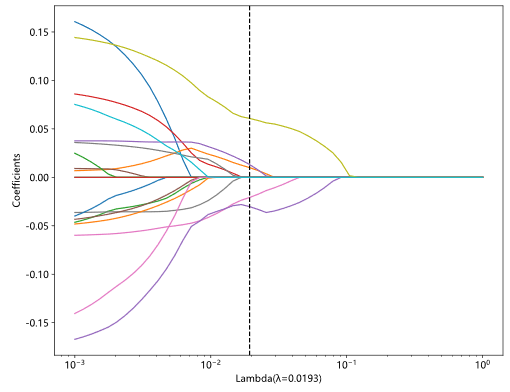


Figure S3. Path plot of lasso coefficients


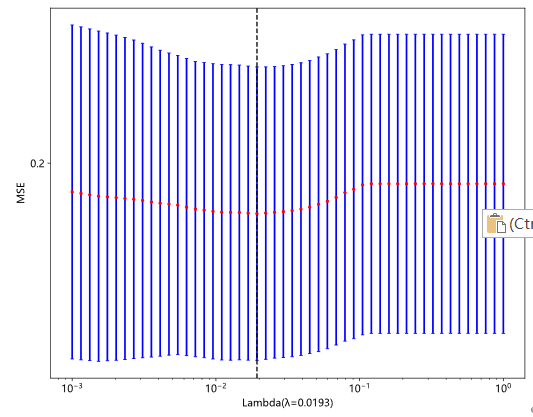


Figure S4. Lasso regression analysis was used for cross-validation


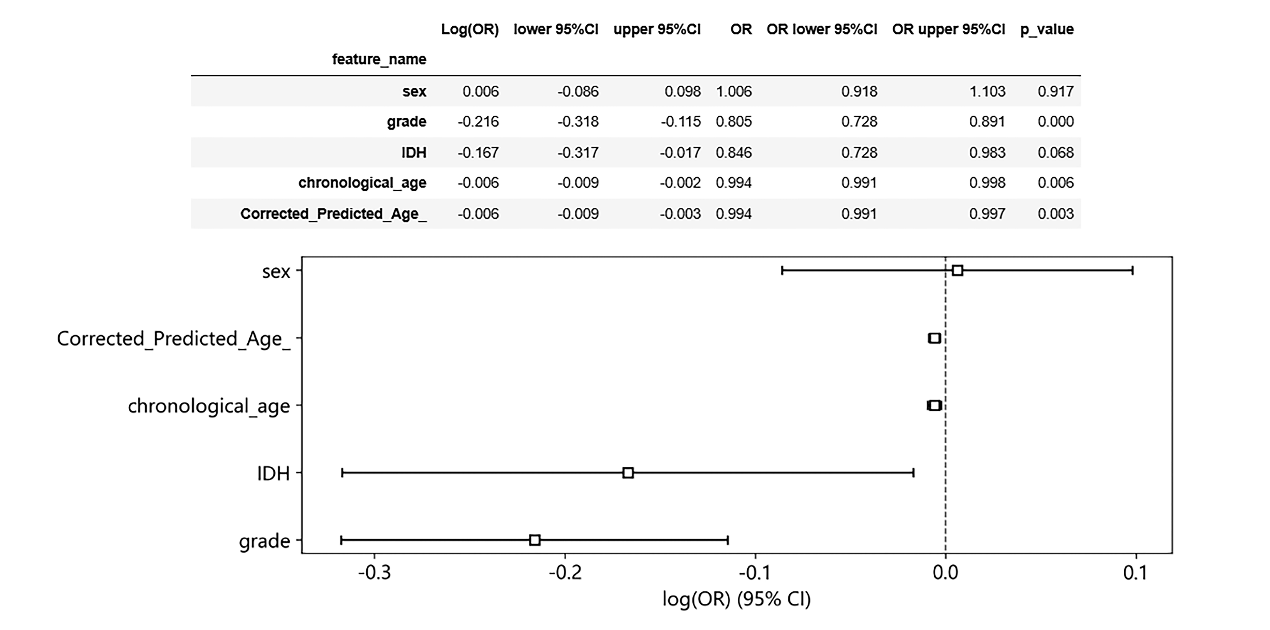
 Figure S5. Clinic multivariate analysis

## **Result**

### **Brain age training model five-fold cross-validation**

We performed internal and external cross-validation of the brain-age trained model. Internal cross-validation was a five-fold cross-validation (Table1), and external cross-validation was multicenter cross-validation (Figure1).

| **Cross-Validation** | | | | | | | | | | |
| --- | --- | --- | --- | --- | --- | --- | --- | --- | --- | --- |
|  | first time | | second time | | third time | | fourth time | | fifth time | |
|  | MAE | STD_err | MAE | STD_err | MAE | STD_err | MAE | STD_err | MAE | STD_err |
| Model1 | 4.7317 | 5.854047 | 4.5899 | 5.6390467 | 4.7379 | 5.8763885 | 4.3252 | 5.085979 | 3.8531 | 4.6816841 |
| Model2 | 3.9154 | 5.545626 | 3.51684 | 5.165115 | 3.541 | 5.16531 | 3.0564 | 5.168351 | 3.21684 | 5.198641 |
| Model3 | 3.6741 | 5.168515 | 3.5729 | 5.374407 | 3.0047 | 4.540597 | 3.4975 | 4.278397 | 3.55125 | 4.316103 |
| Model4 | 4.0974 | 5.1913698 | 4.00022 | 5.1403913 | 4.1184 | 5.2105255 | 4.1498 | 5.3130546 | 4.0233 | 5.13402 |

Table S1 Five-fold cross-validation

MAE is Mean Absolute Error; STD_err is Standard Error

Model1 was trained using all the databases; model2 was trained using the Nanjing brain hospital; model3 was trained using the Yijishan hospital; model4 was trained using the ADNI


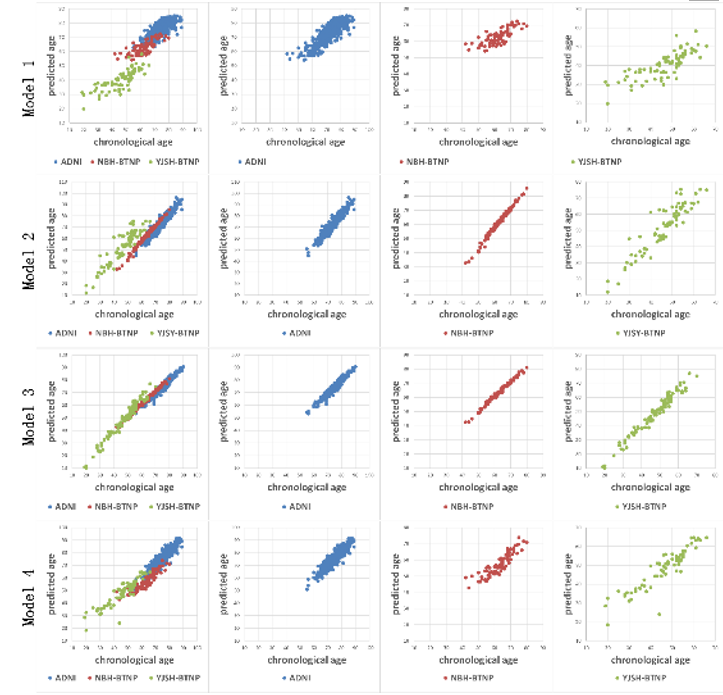


Figure S6. multicenter cross-validation of brain age

Model1 was trained using all the databases; model2 was trained using the Nanjing brain hospital; model3 was trained using the Yijishan hospital; model4 was trained using the ADNI.

### **Whole brain brain age prediction**

We also calculated brain age for patients with glioma with and without masks, and patients without masks performed more stably in the model, which we put in the main text section.


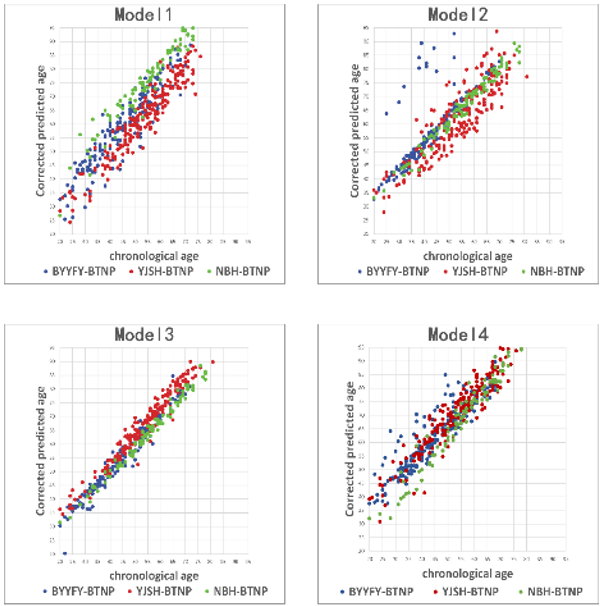


Figure S7. Whole brain glioma brain age prediction (After correction of bias)

Model1 was trained using all the databases; model2 was trained using the Nanjing brain hospital; model3 was trained using the Yijishan hospital; model4 was trained using the ADNI

### **Whole brain glioma with epilepsy brain age**


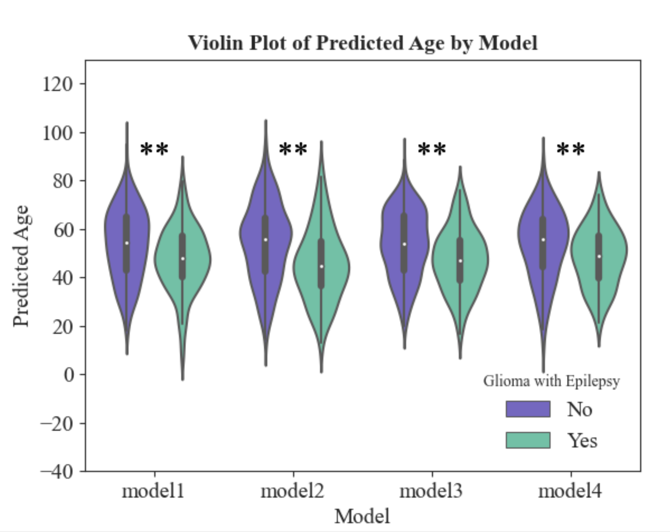


Figure S8. Comparison of brain age between patients with glioma related epilepsy and glioma without epilepsy

* denotes statistically significant p value< 0.05, ** denotes statistically significant p value < 0.01

Model1 was trained using all the databases; model2 was trained using the Nanjing brain hospital; model3 was trained using the Yijishan hospital; model4 was trained using the ADNI

## **References**

1. Li, X., et al., *The first step for neuroimaging data analysis: DICOM to NIfTI conversion.* J Neurosci Methods, 2016. **264**: p. 47-56.

2. Yaniv, Z., et al., *SimpleITK Image-Analysis Notebooks: a Collaborative Environment for Education and Reproducible Research.* J Digit Imaging, 2018. **31**(3): p. 290-303.

3. Smith, S.M., et al., *Advances in functional and structural MR image analysis and implementation as FSL.* Neuroimage, 2004. **23 Suppl 1**: p. S208-19.

4. Yushkevich, P.A., et al., *User-guided 3D active contour segmentation of anatomical structures: significantly improved efficiency and reliability.* Neuroimage, 2006. **31**(3): p. 1116-28.

5. van Griethuysen, J.J.M., et al., *Computational Radiomics System to Decode the Radiographic Phenotype.* Cancer Research, 2017. **77**(21): p. e104-e107.
